# Pediatric Minimally Invasive Surgery—A Bibliometric Study on 30 Years of Research Activity

**DOI:** 10.3390/children9081264

**Published:** 2022-08-21

**Authors:** Boshen Shu, Xiaoyan Feng, Illya Martynov, Martin Lacher, Steffi Mayer

**Affiliations:** Department of Pediatric Surgery, University of Leipzig, 04103 Leipzig, Germany

**Keywords:** bibliometrics, minimally invasive surgery, pediatric surgery

## Abstract

Background: Pediatric minimally invasive surgery (MIS) is a standard technique worldwide. We aimed to analyze the research activity in this field. Methods: Articles on pediatric MIS (1991–2020) were analyzed from the Web of Science™ for the total number of publications, citations, journals, and impact factors (IF). Of these, the 50 most cited publications were evaluated in detail and classified according to the level of evidence (i.e., study design) and topic (i.e., surgical procedure). Results: In total, 4464 publications and 53,111 citations from 684 journals on pediatric MIS were identified. The 50 most cited papers were published from 32 institutions in the USA/Canada (*n* = 28), Europe (*n* = 19), and Asia (*n* = 3) in 12 journals. Four authors (USA/Europe) contributed to 26% of the 50 most cited papers as first/senior author. Hot topics were laparoscopic pyeloplasty (*n* = 9), inguinal hernia repair (*n* = 7), appendectomy, and pyloromyotomy (*n* = 4 each). The majority of publications were retrospective studies (*n* = 33) and case reports (*n* = 6) (IF 5.2 ± 3.2; impact index 16.5 ± 6.4; citations 125 ± 39.4). They were cited as often as articles with high evidence levels (meta-analyses, *n* = 2; randomized controlled trials, *n* = 7; prospective studies, *n* = 2) (IF 12.9 ± 22.5; impact index 14.0 ± 6.5; citations 125 ± 34.7; *p* > 0.05). Conclusions: Publications on laparoscopic pyeloplasty, inguinal hernia repair, appendectomy, and pyloromyotomy are cited most often in pediatric MIS. However, the relevant number of studies with strong evidence for the advantages of MIS in pediatric surgery is missing.

## 1. Introduction

The first pediatric laparoscopic operation was published by Jean-Luc Alain from France in 1991, describing a pyloromyotomy in hypertrophic pyloric stenosis using 3 mm trocars [1,2,3,4]. In the same year, laparoscopic cholecystectomy and ovarian detorsion were reported by George W. Holcomb and Eliezer Shalev [5,6]. The first pediatric thoracoscopic procedure, namely the evacuation of empyema in nine children, was published 2 years later by John A. Kern [7]. In 1999, Thom E. Lobe reported the first thoracoscopic repair of esophageal atresia (Type A) in an 8-month-old infant weighing 3.4 kg [8]. Finally, Klaus Heller described the first robotic fundoplication in 2002 [9].

Since then, minimally invasive surgery (MIS) has been widely accepted for better cosmesis, shorter recovery, less trauma, and better visualization, which are particularly important for infants and adolescents [10,11]. Its success has been documented in numerous case reports, clinical trials, and meta-analyses [12,13,14]. However, until today the research activity on pediatric MIS has not been studied in detail. Bibliometrics estimates the impact of scientific work using mathematical and statistical tools [15]. Research activity can be assessed by publication (quantity) and citation numbers (quality) [16]. Moreover, bibliometric studies can assess individual research interests and enable the identification of potential research collaborations [17].

Here, we aim to analyze the research activity as well as the 50 most cited papers on MIS for their topics as well as evidence levels over the last 30 years. We hypothesized that the trend of research activity as well as the evidence levels of publications on pediatric MIS has been increasing over time.

## 2. Materials and Methods

Original, peer-reviewed scientific publications published on pediatric MIS between 1991 and 2020 were identified using the Web of Science Core Collection™ (www.webofknowledge.com, Clarivate Analytics, Boston, MA, USA) by two independent reviewers (BS, XF) on 1 March 2021 according to the search items listed in Table 1. These inclusion and exclusion items were defined by the research group to allow the identification of as many and specific publications on pediatric MIS as possible. Additionally, to analyze only relevant search results, a “title” instead of “topic” search approach was used [16].

All identified articles reporting on minimally invasive interventions in children identified by this search were screened for the study. Papers reporting on diagnostic interventions only, e.g., diagnostic laparoscopy or endoscopy, or from other surgical fields such as neurosurgery or cardiac surgery were excluded from our dataset. There were no restrictions on the type of article or language.

Data on publications extracted from the Web of Science^TM^ software included: publication year, country/continent, institution, author, and journal. Number of publications defined the particular research quantity. Research quality was defined as the total number of citations and impact index as well as impact factor of the corresponding journal. The impact factor was extracted from the Journal Citation Reports (Clarivate Analytics) for 2020. The impact index was calculated by dividing the number of years since publication by the number of citations and then multiplied by 100. The lower the impact index, the higher the citation rate since publication, thus indicating an augmented recognition [16].

To identify hot topics of pediatric MIS research, the 50 most cited papers were examined in detail. At first, the top 10 institutions, first/senior authors, and journals defined by the number of publications were recorded. Second, papers were screened manually by two independent authors (BS, XF) for the disease and/or operative procedure such as thoracoscopy, laparoscopy, thoracic operations, gastrointestinal or urological surgery. Evidence levels were classified according to Cashin et al. from high to low: meta-analyses (Level I), randomized controlled trials (RCTs) (Level I), prospective studies (Level II), retrospective studies (Level III), and case reports (Level IV) [18]. Level I and II were defined as high evidence levels.

Statistical analyses were performed with GraphPad Prism v. 7.0 (GraphPad, La Jolla, CA, USA). All tests were two-sided. The Spearman correlation coefficient was used to test correlations between selected continuous variables. Unpaired *t* tests were used to compare two different groups for parametric data and the Wilcoxon test was used for non-parametric data. *p*-Values of <0.05 were considered statistically significant. Visualized analysis for country collaboration of the top 50 cited articles was performed using VOSviewer 1.6.16 (Leiden University, Leiden, The Netherlands). Here, the line thickness between the colored dots indicates the total link strength, while the size of dots represents the number of publications in bibliographic coupling.

## 3. Results

### 3.1. Overall Trends

**Figure 1:**
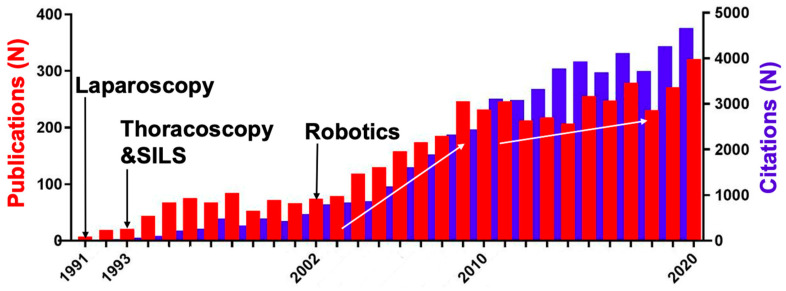
Publication and citation trends on pediatric MIS between 1991 and 2020. The first pediatric laparoscopic, single-incision laparoscopic surgery (SILS), thoracoscopic, and robotic interventions were published in 1991, 1993, and 2002, respectively. The number of publications (red) and citations (blue) significantly increased over time, with the steepest increase between 2002 and 2009.

A total of 4464 publications and 53,111 citations from 684 journals on pediatric MIS between 1991 and 2020 were included in the analysis. The first pediatric laparoscopic, SILS (single-incision laparoscopic surgery), thoracoscopic, and robotic operations were published in 1991, 1993, and 2002, respectively [1,2,7,9,19]. The number of publications and citations per year constantly increased from 1991 to 2020, from seven and three, respectively, to 321 and 4666 in a similar matter (r = 0.96, *p* < 0.001), with the steepest increase between 2002 and 2009 (Figure 1). The number of publications correlated well with the number of citations during the last 30 years (r = 0.91, *p* < 0.0001).

### 3.2. 50 Most Cited Publications on MIS

**Figure 2:**
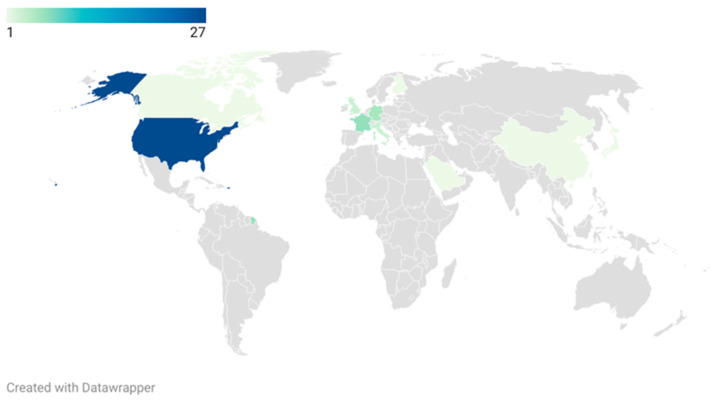
Choropleth map depicting the geographical distribution of the top 50 cited publications on pediatric MIS.

**Figure 3:**
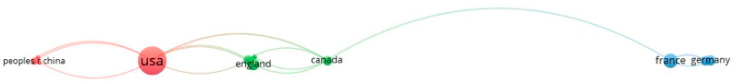
Country-wise co-authorship collaborations of the top 50 cited publications on pediatric MIS.

The 50 most cited manuscripts were published between 1991 and 2013 and derived from 32 institutions in North America (*n* = 28), Europe (*n* = 19), and Asia (*n* = 3), as listed in Table 2. The United States of America holds the majority (27/50) of the global publication pattern (Figure 2) as well as the leading position in country-wise collaboration, owning eight total link strengths (Figure 3). The number of total citations ranged from 90 to 221 per paper (mean: 125 ± 38.1), with an average impact index of 15.9 ± 6.4. The most often cited article was published in 2006 by Richard S. Lee in the *Journal of Urology* (impact index: 6.8, IF: 7.5), comparing the safety and efficacy between robotic-assisted laparoscopic and open pyeloplasty in children, which showed comparable safety but longer operation time for the robotic procedure [20]. The second most often cited publication was from Keith E. Georgeson, published in 2000 in the *Journal of Pediatric Surgery* (impact index: 9.1, IF: 1.9), describing the laparoscopically-assisted anorectal pull-through (LAARP) as a new technique for the repair of high imperforate anus. The authors reported an excellent visualization of the rectal fistula and surrounding structures, accurate placement of the bowel through the anatomic midline and levator sling, and minimally invasive abdominal and perineal wounds [21].

### 3.3. Top Cited Journals and Impact Factor

The 50 most cited manuscripts were published in 12 journals with an IF ranging from 1.4 to 79.3. The *Journal of Pediatric Surgery* (*n* = 17; 34%; IF = 2.5), *Journal of Urology* (*n* = 12; 24%; IF = 7.5), and *Annals of Surgery* (*n* = 6; 12%; IF = 13.0) hosted 70% of the top cited papers. More than 50% of the top 50 citations were published with an IF > 2.5, and 14% with an IF > 10. The publication with the highest IF (*The Lancet*, IF = 79.3) was an RCT by Nigel J. Hall from 2009 reporting the outcome of open versus laparoscopic pyloromyotomy, indicating that both procedures were equally safe [22]. Moreover, the requirement of analgesics was significantly higher and parental satisfaction significantly lower after the open procedure. Thus, the authors recommended the minimally invasive approach in centers with sufficient laparoscopic experience.

### 3.4. Evidence Levels

**Figure 4:**
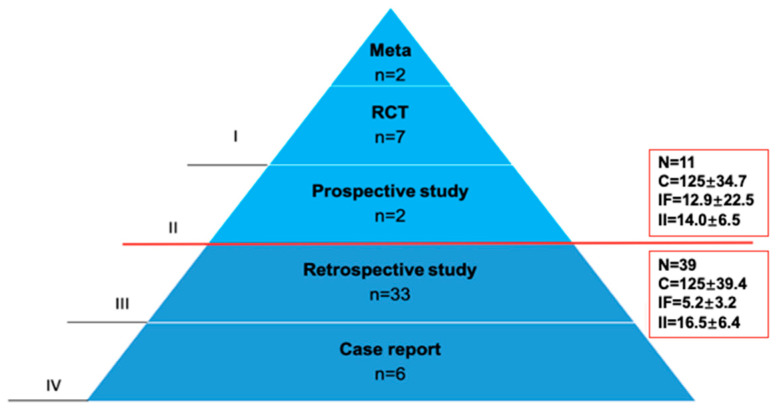
Evidence levels of the top 50 cited papers in pediatric MIS (1991 to 2020). The minority of manuscripts provided high evidence (*n* = 11; level I/II) and was published at a comparable mean citation rate and impact index as the 39 papers with lower evidence level (*p* > 0.05). Meta: meta-analysis; RCT: randomized controlled trial; N: total number of publications; C: total number of citations; IF: impact factor; II: impact index.

**Figure 5:**
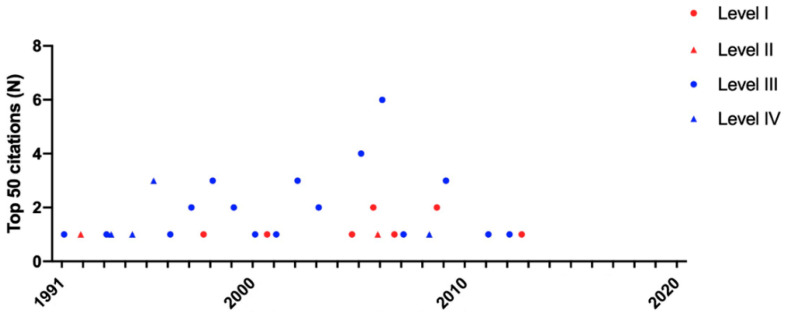
Evidence levels of the 50 most cited papers on pediatric MIS (1991 to 2020). The top 50 cited papers were published from 1991 to 2013. Manuscripts of high- (level I/II; *red)* and low-evidence level (level III/IV; *blue)* were distributed equally over time.

The majority of the top 50 citations were retrospective studies (Level III; 66%) and case reports (Level IV; 12%), while the minority were published with high levels of evidence (I/II; 22%) (Figure 4). As a result, retrospective studies (Level III) and case reports (Level IV) accounted for more than 75% of the top 50 citations on pediatric MIS (Figure 5). Neither the IF (12.9 ± 22.5 vs. 5.2 ± 3.2; *p* = 0.46) nor the average number of citations (*n* = 125 ± 39.4 vs. *n* = 125 ± 34.7; *p* = 0.63) or mean impact index (14.0 ± 6.5 vs. 16.5 ± 6.4; *p* = 0.20) of high- and low-evidence level studies differed significantly.

### 3.5. Hot Topics

**Figure 6:**
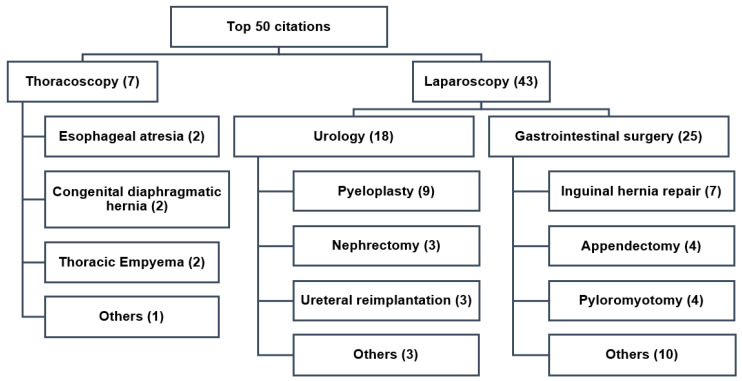
Hot topics of the 50 most cited papers on pediatric MIS (1991 to 2020). The majority of papers reported on laparoscopy (*n* = 43; 86%), with urologic interventions playing an important role (*n* = 18; 36%) in contrast to thoracoscopic procedures (*n* = 7; 14%).

The majority of the 50 most cited papers reported on laparoscopic procedures (86%) (Figure 6; Table 2 and Table 3). Minimally invasive inguinal hernia repair (14%), appendectomy (8%), and pyloromyotomy (8%) dominated gastrointestinal interventions (50%). Pyeloplasty (18%), nephrectomy (6%), and ureteral reimplantation (6%) directed urological procedures (36%). Thoracoscopy was underrepresented (14%) and reported on the minimally invasive treatment of esophageal atresia (4%), congenital diaphragmatic hernia (4%), and empyema (4%).

## 4. Discussion

The absolute number of publications and citations on pediatric MIS increased during the last 30 years, with a steep rise between 2002 and 2009. This is in line with publications on other pediatric surgical topics such as esophageal atresia, anorectal malformations, and biliary atresia, and can be explained by the enhanced ambitions to share medical findings with the research community [16,23,24]. Moreover, research activity represents one of the most important factors to rate one’s academic value. Accordingly, a higher h-index correlates with a higher academic faculty rank [25].

In total, 32 institutions from three continents contributed to the 50 most cited articles on pediatric MIS. North America provided the majority of citations, which was also seen in other research topics such as neurocritical care and meniscal injury [26,27]. Additionally, the United States of America had the leading position in co-authorship country-wise collaborations and contributed the largest number of publications. This might be explained by the impact of science and technology budgets of this country and the financial support of organizations [28,29,30]. In general, countries with a high-income society accomplish more output of their research, while low- and middle-income countries publish relatively less scientific work [31].

### 4.1. Scientific Quality of the Top 50 Citations

The impact factor of the top 50 citations ranged from 1.4 to 79.3, with more than half of the manuscripts published in journals with an impact factor above 2.5, which equals the highest impact factor of pediatric surgery specific journals, i.e., the Journal of Pediatric Surgery. Thus, the majority of top 50 citations were published in non-pediatric surgery journals. This is in line with other research fields such as oncology, reporting that top cited papers are preferentially published in high-impact journals [32]. One may speculate that the most cited papers have profound influence on clinical practice or future developments also beyond a specific research field and are therefore published in more generalized journals with higher impact factors due to a broader audience [32]. Conversely, papers from journals with higher IF are preferentially cited, which may induce a publication bias [33].

The evidence level of a published study may be a superior quality parameter of the scientific work [34]. In the top 50 citations on pediatric MIS, studies with high impact, i.e., meta-analyses, RCTs, and prospective trials, were underrepresented. One meta-analysis summarized 23 studies on 6477 appendectomies, reporting lower rates of wound infection and ileus, shorter postoperative stay, as well as comparable operative time and complications for the laparoscopic approach [14]. The other one investigated the laparoscopic diagnosis of inguinal hernia [35]. The majority (78%) of the top 50 citations were retrospective studies and case reports, which is comparable to other bibliometric studies such as in orthopedic surgery [36,37]. Both approaches are important to investigate rare diseases, manifestations, and outcomes. However, their scientific value is limited: Some information may be missing, selection and recall biases can affect the results, and reasons for differences in treatment or loss of follow-ups can often not be ascertained [38]. Nevertheless, retrospective studies and also case reports require less time and lower budgets and can pave the way to define new research questions and prospective trials [39]. However, to underline the advantages of MIS in pediatric surgery, prospective and RCT trials, as the gold standard of effective research, are required [40].

### 4.2. Hot Topics of the Top 50 Citations

Being the earliest established and widely performed approach, laparoscopic interventions dominated the top 50 citations in our bibliometric study on pediatric MIS, while thoracoscopic interventions were less common. In the subgroup of abdominal and urologic interventions, minimally invasive pyeloplasty and inguinal hernia repair accounted for one-third of the top 50 citations.

Ureteropelvic junction obstruction (UPJO) occurs in 1 per 1.000–2.000 newborns, of which 10–20% undergo surgery later in life [41,42]. In 1993, Tan and his team reported on the first six children with UPJO treated by laparoscopic pyeloplasty. Five of them had normal or significantly improved drainage times postoperatively [43].

Nowadays, the laparoscopic approach is comparable to the open procedure with regards to safety and effectiveness [44]. However, despite similar complication rates and shorter lengths of stay, especially in children ≤ 2 years, UPJO is treated only in 25% of German patients laparoscopically. This could be explained by the challenging surgical technique as well as the low utilization of laparoscopy in non-teaching hospitals in Germany [45,46,47].

The cumulative incidence of inguinal hernia before the age of 15 is up to 7% in males and 1% in females [48]. Since the first laparoscopic inguinal hernia was reported by Montupet et al. in 1993, various minimally invasive procedures have been published [49]. Nowadays, the extraperitoneal approach is preferred worldwide [50]. According to a guideline of the European Pediatric Surgeon’s Association in 2022, laparoscopic inguinal hernia repair is beneficial for children with bilateral hernia, incarceration, and recurrence [51,52,53,54,55]. Accordingly, a recent systematic review on 13 RCTs reported on a shorter operative time for bilateral hernias, fewer post-operative complications and metachronous inguinal hernia rates for laparoscopic herniorrhaphy. No significant differences were found for unilateral operative time, time to full recovery, length of hospital stay, recurrence, and postoperative hydrocele [56].

In contrast to laparoscopy, thoracoscopic procedures accounted for only 14% of the top 50 citations on pediatric MIS. These papers mainly reported on neonatal thoracoscopy in EA, CDH, and thoracic empyema. The incidences of these diseases are low, and the surgical skills required to carry out these procedures are much higher than for routine laparoscopy [57,58,59]. Similarly, thoracoscopic MIS faces more obstacles of limited space, demanding anesthesia, and specialized instruments, especially in neonates [60]. Although the first thoracoscopic intervention for acute empyema was described as early as 1993, the first thoracoscopic procedures in CDH, EA, and congenital lung malformations were reported almost one decade later [7,60,61,62].

### 4.3. Establishing New Techniques in Pediatric MIS

When establishing a new technique in (MIS) surgery, different aspects need to be taken into account. First, the incidence of the disease should be high enough to pass your learning curve quickly. Second, the intervention itself should be well-defined and not exceed your surgical skills. Third, in case of technical difficulties, conversion to open surgery needs to be easy to prevent harm to the patient. Similarly, the conversion rate is an important parameter when evaluating new MIS procedures. Based on a German nationwide cohort study published in 2016, 75% of pediatric appendectomies were performed minimally invasively with a conversion rate of 1.2% [63]. In contrast, the reported conversion rate of thoracoscopic CDH and EA repair can be as high as 33–75% [64,65]. Technical difficulties, but also the effects of increased abdominal pressure, intraoperative hypercapnia, and acidosis, may contribute to the higher conversion rate in those cases [66].

The establishment of a new technique also depends on technical refinements, experience, individual learning curves, as well as the growing number of patients operated upon, i.e., experience, as well as results published. Most learning curve studies report a significant decrease in operative time as well as perioperative and postoperative complications with increasing experience of the surgeon [67]. The number of procedures a surgeon needs to pass his/her learning curve for perioperative and postoperative complications, recurrences, and conversion rates varies in different interventions. Similarly, one should perform 30, 20, 51, and 37 cases of laparoscopic inguinal hernia repair, laparoscopic pyloromyotomy, laparoscopic appendectomy, and robotic-assisted pyeloplasty, respectively, to get over his/her learning curve [68,69,70,71]. Moreover, experienced surgeons have lower complication rates and need to perform fewer cases to reach their plateau [68].

Although the first SILS and robotic-assisted interventions in children were published as early as in 1993 and 2002, respectively, none of the top 50 citations published on pediatric robotics or SILS [19]. This underlines the results from a survey among International Pediatric Endosurgery Group (IPEG) members stating that 80% perform SILS for cases of lower complexity such as appendectomy, although 70% of respondents find the scientific evidence for the benefits of SILS is not convincing [72].

Robotic interventions also require advanced surgical skills as well as appropriate equipment. Similarly, the first robotic surgery in a child, a Nissen fundoplication, was published almost 10 years after the first adult cases, and the spread of this new technique is relatively slower than that of other MIS techniques [73,74].

### 4.4. Limitations

Our study has several limitations. At first, only the Web of Science™ database was used to search for publications, thus, other sources may have led to a different number of research items or citation counts. Second, we aimed to identify only related articles on pediatric MIS, thus “title” instead of “topic” searching strategy was used. This might exclude some, but most likely an insignificant number of related articles. Finally, bibliometric studies always reflect the current state of the literature at the time of analysis and cannot rule out the impact of time with new publications and citations.

## 5. Conclusions

Research activity on pediatric MIS increased over the last 30 years, with a golden decade in the early 21st century. Laparoscopic pyeloplasty and inguinal hernia repair accounted for most of the top 50 citations. Retrospective trials and case reports dominated the evidence circulated. Studies with strong evidence are missing, especially on advanced techniques in pediatric MIS.

## Figures and Tables

**Table 1:** Inclusion and exclusion items of the Web of Science search.

“thoracoscopy” OR “thoracoscopic” OR “thoracoscopically” OR “laparoscopy” OR “laparoscopic” OR “laparoscopically” OR “minimal invasive surgery” OR “minimally invasive surgery” OR “robot assisted”
AND
“neonate” OR “neonates” OR “neonatal” OR “infant” OR “infants” OR “infancy” OR “preterm” OR “preterms” OR “newborn” OR “newborns” OR “pediatric” OR “pediatrics” OR “children” OR “child” OR “boy” OR “girl” OR “boys” OR “girls” OR “adolescent” OR “congenital” OR “atresia” OR “tracheoesophageal fistula” OR “necrotizing enterocolitis” OR “Hirschsprung disease” OR “anorectal malformation” OR “neuroblastoma” OR “hepatoblastoma” OR “nephroblastoma” OR “wilms” OR “orchidopexy” OR “pyloromyotomy” OR “Kasai” OR “imperforate anus”
NOT
“CHD” OR “patent ductus arteriosus” OR “PDA” OR “neurosurgery” OR “thalamic astrocytomas” OR “GAIT” OR “Palsy” OR “stereoelectroencephalography” OR “ASD” OR “Autism” OR “brain” OR “brainstem” OR “neuromotor” OR “attention-deficit hyperactivity disorder” OR “ADHD” OR “idiopathic scoliosis” OR “spinal” OR “spine”

Key words were selected for age of patients, specific MIS procedures, and MIS-specific diseases in infants, excluding congenital heart disease and skeletal, neurologic, and mental diseases.

**Table 2:** 50 most cited publications on pediatric MIS between 1991 and 2020 sorted by number of citations.

	Publication First Author	Journal	Total Citations (n)	Year	Impact Index	Impact Factor (2020)	Evidence Level	Country
1	*Pediatric robot assisted laparoscopic dismembered pyeloplasty: Comparison with a cohort of open surgery*							
	Lee RS	J Urol	221	2006	6.3	5.9	retrospective	USA
2	*Laparoscopically assisted anorectal pull-through for high imperforate anus—A new technique*							
	Georgeson KE	J Pediatr Surg	220	2000	9.1	1.9	retrospective	USA
3	*Primary laparoscopic pull-through for hirschsprungs-disease in infants and children*							
	Georgeson KE	J Pediatr Surg	212	1995	11.8	1.9	case report	USA
4	*Laparoscopic versus open appendectomy in children—A meta-analysis*							
	Aziz O	Ann Surg	205	2006	6.8	10.1	meta analysis	UK
5	*Pediatric laparoscopic dismembered pyeloplasty*							
	Peters CA	J Urol	193	1995	13.0	5.9	case report	USA
6	*Single-port laparoscopic surgery: initial experience in children for varicocelectomy*							
	Kaouk JH	BJU Int	182	2008	6.6	4.8	case report	USA
7	*Thoracoscopic repair of esophageal atresia and tracheoesophageal fistula—A multi-institutional analysis*							
	Holcomb GW	Ann Surg	182	2005	8.2	10.1	retrospective	USA
8	*Congenital choledochal cyst: video-guided laparoscopic treatment*							
	Farello GA	Surg Laparosc Endosc	167	1995	15.0	1.4	case report	Italy
9	*Laparoscopic inguinal herniorrhaphy in children: A three-center experience with 933 repairs*							
	Schier F	J Pediatr Surg	164	2002	11.0	1.9	retrospective	Germany
10	*Thoracoscopic decortication vs. tube thoracostomy with fibrinolysis for empyema in children: a prospective, randomized trial*							
	St Peter SD	J Pediatr Surg	157	2009	7.0	1.9	RCT	USA
11	*Laparoscopic inguinal hernia repair—a prospective personal series of 542 children*							
	Schier F	J Pediatr Surg	155	2006	9.0	1.9	prospective	Germany
12	*Laparoscopic Anderson-Hynes dismembered pyeloplasty in children*							
	Tan HL	J Urol	151	1999	13.9	5.9	retrospective	UK
13	*Laparoscopic percutaneous extraperitoneal closure for inguinal hernia in children: clinical outcome of 972 repairs done in 3 pediatric surgical institutions*							
	Takehara H	J Pediatr Surg	149	2006	9.4	1.9	retrospective	Japan
14	*Early Experience with Single-Port Laparoscopic Surgery in Children*							
	Ponsky TA	J Laparoendosc Adv Surg Tech A	141	2009	7.8	1.4	retrospective	USA
15	*Laparoscopic vesicoureteroplasty in children: initial case reports*							
	Ehrlich RM	Urology	136	1994	19.1	1.9	case report	USA
16	*A multi-institutional analysis of laparoscopic orchidopexy*							
	Baker LA	BJU Int	129	2001	14.7	4.8	retrospective	USA
17	*Laparoscopic treatment of congenital inguinal hernia in children*							
	Montupet P	J Pediatr Surg	126	1999	16.7	1.9	retrospective	Italy
18	*Open versus laparoscopic pyloromyotomy for pyloric stenosis—A prospective, randomized trial*							
	St Peter SD	Ann Surg	124	2006	11.3	10.1	RCT	USA
19	*Prospective, randomized, single-center, single-blind comparison of laparoscopic vs. open repair of pediatric inguinal hernia*							
	Chan KL	Surg Endosc	123	2005	12.2	3.1	RCT	Peoples R China
20	*Initial comparison of robotic-assisted laparoscopic versus open pyeloplasty in children*							
	Yee DS	Urology	120	2006	11.7	1.9	retrospective	USA
21	*Recovery after open versus laparoscopic pyloromyotomy for pyloric stenosis: A double-blind multicentre randomised controlled trial*							
	Hall NJ	Lancet	113	2009	9.7	60.4	RCT	UK
22	*Retroperitoneal laparoscopic versus open pyeloplasty in children*							
	Bonnard A	J Urol	112	2005	13.4	6	retrospective	France
23	*Laparoscopic Sleeve Gastrectomy in 108 Obese Children and Adolescents Aged 5 to 21 Years*							
	Alqahtani AR	Ann Surg	111	2012	7.2	10.1	retrospective	Saudi Arabia
24	*Experience with 220 consecutive laparoscopic Nissen fundoplications in infants and children*							
	Rothenberg SS	J Pediatr Surg	110	1998	20.0	1.9	retrospective	USA
25	*Laparoscopic renal surgery via a retroperitoneal approach in children*							
	El-Ghoneimi A	J Urol	110	1998	20.0	5.9	retrospective	France
26	*Is there a role for laparoscopic appendectomy in pediatric surgery?*							
	Gilchrist BF	J Pediatr Surg	109	1992	25.7	1.9	prospective	USA
27	*Thoracoscopic repair of tracheoesophageal fistula in newborns*							
	Rothenberg SS	J Pediatr Surg	108	2002	16.7	1.9	retrospective	USA
28	*Laparoscopic dismembered pyeloplasty by a retroperitoneal approach in children*							
	El-Ghoneimi A	BJU Int	108	2003	15.7	4.8	retrospective	France
29	*Robotic assisted laparoscopic pyeloplasty in children*							
	Atug F	J Urol	108	2005	13.9	5.9	retrospective	USA
30	*Laparoscopic evaluation of the pediatric inguinal hernia—A meta-analysis*							
	Miltenburg DW	J Pediatr Surg	109	1998	21.0	1.9	meta analysis	USA
31	*Pediatric laparoscopic splenectomy*							
	Tulman S	J Pediatr Surg	103	1993	26.2	1.9	case report	USA
32	*Thoracoscopy in the management of empyema in children*							
	Kern JA	J Pediatr Surg	103	1993	26.2	1.9	retrospective	USA
33	*Robotic Assisted Laparoscopic Ureteral Reimplantation in Children: Case Matched Comparative Study With Open Surgical Approach*							
	Marchini Giovanni S	J Urol	101	2011	8.9	5.9	retrospective	USA
34	*Laparoscopic splenic procedures in children—Experience in 231 children*							
	Rescorla FJ	Ann Surg	100	2007	13.0	10.1	retrospective	USA
35	*Thoracoscopy Versus Thoracotomy Improves Midterm Musculoskeletal Status and Cosmesis in Infants and Children*							
	Lawal Taiwo A	Ann Thorac Surg	100	2009	11.0	3.6	retrospective	Germany
36	*Laparoscopic heminephroureterectomy in pediatric patients*							
	Janetschek G	J Urol	100	1997	23.0	5.9	retrospective	Austria
37	*Laparoscopic transabdominal pyeloplasty in children is feasible irrespective of age*							
	Metzelder ML	J Urol	99	2006	14.1	5.9	retrospective	Germany
38	*Hypercapnia and Acidosis During Open and Thoracoscopic Repair of Congenital Diaphragmatic Hernia and Esophageal Atresia Results of a Pilot Randomized Controlled Trial*							
	Bishay M	Ann Surg	97	2013	7.2	10.1	RCT	Canada
39	*Neonatal thoracoscopic repair of congenital diaphragmatic hernia: Selection criteria for successful outcome*							
	Yang EY	J Pediatr Surg	95	2005	15.8	1.9	retrospective	USA
40	*Extramucosal pyloromyotomy by laparoscopy*							
	Alain JL	Surg Endosc	94	1991	30.9	3.1	retrospective	France
41	*Retroperitoneal laparoscopic vs. open partial nephroureterectomy in children*							
	El-Ghoneimi A	BJU Int	93	2003	18.3	4.8	retrospective	France
42	*Laparoscopic pyloromyotomy for hypertrophic pyloric stenosis: A prospective, randomized controlled trial*							
	Leclair MD	J Pediatr Surg	92	2007	14.1	1.9	RCT	France
43	*Single-blind randomized clinical trial of laparoscopic versus open appendicectomy in children*							
	Lintula H	Br J Surg	91	2001	24.2	5.7	RCT	Finland
44	*Laparoscopic herniorrhaphy in girls*							
	Schier F	J Pediatr Surg	91	1998	20.9	1.9	retrospective	Germany
45	*One-trocar transumbilical laparoscopic-assisted appendectomy in children: Our experience*							
	D’Alessio A	Eur J Pediatr Surg	91	2002	19.8	2.3	retrospective	Italy
46	*Experience with Modified Single-Port Laparoscopic Procedures in Children*							
	Rothenberg SS	J Laparoendosc Adv Surg Tech A	90	2009	12.2	1.4	retrospective	USA
47	*Complications in pediatric urological laparoscopy: Results of a survey*							
	Peters CA	J Urol	90	1996	26.7	5.9	retrospective	USA
48	*Laparoscopic pyeloplasty in the infant younger than 6 months—Is it technically possible?*							
	Kutikov A	J Urol	90	2006	15.6	5.9	retrospective	USA
49	*Initial experience with laparoscopic transvesical ureteral reimplantation at the Children’s Hospital of Philadelphia*							
	Kutikov A	J Urol	90	2006	15.6	5.9	retrospective	USA
50	*Should laparoscopic appendectomy be avoided for complicated appendicitis in children?*							
	Horwitz JR	J Pediatr Surg	90	1997	25.6	1.9	retrospective	USA

**Table 3:** 50 most cited publications on pediatric MIS between 1991 and 2020 sorted by impact index. The lower the impact index, the higher the citation rate since publication, thus indicating an augmented recognition.

	*Publication* First Author	Journal	Total Citations (*n*)	Year	Impact Index	Impact Factor (2020)	Evidence Level	Country
1	*Pediatric Robot Assisted Laparoscopic Dismembered Pyeloplasty: Comparison with a Cohort of Open Surgery*							
	Lee RS	J Urol	221	2006	6.3	5.9	retrospective	USA
2	*Single-Port Laparoscopic Surgery: Initial Experience in Children for Varicocelectomy*							
	Kaouk JH	BJU Int	182	2008	6.6	4.8	case report	USA
3	*Laparoscopic Versus Open Appendectomy in Children—A Meta-Analysis*							
	Aziz O	Ann Surg	205	2006	6.8	10.1	meta-analysis	UK
4	*Thoracoscopic Decortication Vs. Tube Thoracostomy with Fibrinolysis for Empyema in Children: A Prospective, Randomized Trial*							
	St Peter SD	J Pediatr Surg	157	2009	7	1.9	RCT	USA
5	*Laparoscopic Sleeve Gastrectomy in 108 Obese Children and Adolescents Aged 5 to 21 Years*							
	Alqahtani AR	Ann Surg	111	2012	7.2	10.1	retrospective	Saudi Arabia
6	*Hypercapnia and Acidosis During Open and Thoracoscopic Repair of Congenital Diaphragmatic Hernia and Esophageal Atresia Results of a Pilot Randomized Controlled Trial*							
	Bishay M	Ann Surg	97	2013	7.2	10.1	RCT	Canada
7	*Early Experience with Single-Port Laparoscopic Surgery in Children*							
	Ponsky TA	J Laparoendosc Adv Surg Tech A	141	2009	7.8	1.4	retrospective	USA
8	*Thoracoscopic Repair of Esophageal Atresia and Tracheoesophageal Fistula—A Multi-Institutional Analysis*							
	Holcomb GW	Ann Surg	182	2005	8.2	10.1	retrospective	USA
9	*Robotic Assisted Laparoscopic Ureteral Reimplantation in Children: Case Matched Comparative Study with Open Surgical Approach*							
	Marchini Giovanni S	J Urol	101	2011	8.9	5.9	retrospective	USA
10	*Laparoscopic Inguinal Hernia Repair—A Prospective Personal Series of 542 Children*							
	Schier F	J Pediatr Surg	155	2006	9	1.9	prospective	Germany
11	*Laparoscopically Assisted Anorectal Pull-Through for High Imperforate Anus—A New Technique*							
	Georgeson KE	J Pediatr Surg	220	2000	9.1	1.9	retrospective	USA
12	*Laparoscopic Percutaneous Extraperitoneal Closure for Inguinal Hernia in Children: Clinical Outcome Of 972 Repairs Done In 3 Pediatric Surgical Institutions*							
	Takehara H	J Pediatr Surg	149	2006	9.4	1.9	retrospective	Japan
13	*Recovery After Open Versus Laparoscopic Pyloromyotomy for Pyloric Stenosis: A Double-Blind Multicentre Randomised Controlled Trial*							
	Hall NJ	Lancet	113	2009	9.7	60.4	RCT	UK
14	*Laparoscopic Inguinal Herniorrhaphy In Children: A Three-Center Experience With 933 Repairs*							
	Schier F	J Pediatr Surg	164	2002	11	1.9	retrospective	Germany
15	*Thoracoscopy Versus Thoracotomy Improves Midterm Musculoskeletal Status and Cosmesis in Infants And Children*							
	Lawal Taiwo A	Ann Thorac Surg	100	2009	11	3.6	retrospective	Germany
16	*Open Versus Laparoscopic Pyloromyotomy for Pyloric Stenosis—A Prospective, Randomized Trial*							
	St Peter SD	Ann Surg	124	2006	11.3	10.1	RCT	USA
17	*Initial Comparison Of Robotic-Assisted Laparoscopic Versus Open Pyeloplasty in Children*							
	Yee DS	Urology	120	2006	11.7	1.9	retrospective	USA
18	*Primary Laparoscopic Pull-Through for Hirschsprungs-Disease In Infants and Children*							
	Georgeson KE	J Pediatr Surg	212	1995	11.8	1.9	case report	USA
19	*Prospective, Randomized, Single-Center, Single-Blind Comparison of Laparoscopic Vs. Open Repair of Pediatric Inguinal Hernia*							
	Chan KL	Surg Endosc	123	2005	12.2	3.1	RCT	Peoples R China
20	*Experience with Modified Single-Port Laparoscopic Procedures in Children*							
	Rothenberg SS	J Laparoendosc Adv Surg Tech A	90	2009	12.2	1.4	retrospective	USA
21	*Pediatric Laparoscopic Dismembered Pyeloplasty*							
	Peters CA	J Urol	193	1995	13	5.9	case report	USA
22	*Laparoscopic Splenic Procedures in Children—Experience in 231 Children*							
	Rescorla FJ	Ann Surg	100	2007	13	10.1	retrospective	USA
23	*Retroperitoneal Laparoscopic Versus Open Pyeloplasty in Children*							
	Bonnard A	J Urol	112	2005	13.4	6	retrospective	France
24	*Laparoscopic Anderson-Hynes Dismembered Pyeloplasty in Children*							
	Tan HL	J Urol	151	1999	13.9	5.9	retrospective	UK
25	*Robotic Assisted Laparoscopic Pyeloplasty in Children*							
	Atug F	J Urol	108	2005	13.9	5.9	retrospective	USA
26	*Laparoscopic Transabdominal Pyeloplasty in Children Is Feasible Irrespective of Age*							
	Metzelder ML	J Urol	99	2006	14.1	5.9	retrospective	Germany
27	*Laparoscopic Pyloromyotomy for Hypertrophic Pyloric Stenosis: A Prospective, Randomized Controlled Trial*							
	Leclair MD	J Pediatr Surg	92	2007	14.1	1.9	RCT	France
28	*A Multi-Institutional Analysis of Laparoscopic Orchidopexy*							
	Baker LA	BJU Int	129	2001	14.7	4.8	retrospective	USA
29	*Congenital Choledochal Cyst: Video-Guided Laparoscopic Treatment*							
	Farello GA	Surg Laparosc Endosc	167	1995	15	1.4	case report	Italy
30	*Laparoscopic pyeloplasty in the infant younger than 6 months—Is it technically possible?*							
	Kutikov A	J Urol	90	2006	15.6	5.9	retrospective	USA
31	*Initial experience with laparoscopic transvesical ureteral reimplantation at the Children’s Hospital of Philadelphia*							
	Kutikov A	J Urol	90	2006	15.6	5.9	retrospective	USA
32	*Laparoscopic Dismembered Pyeloplasty by a Retroperitoneal Approach in Children*							
	El-Ghoneimi A	BJU Int	108	2003	15.7	4.8	retrospective	France
33	*Neonatal Thoracoscopic Repair of Congenital Diaphragmatic Hernia: Selection Criteria for Successful Outcome*							
	Yang EY	J Pediatr Surg	95	2005	15.8	1.9	retrospective	USA
34	*Laparoscopic Treatment of Congenital Inguinal Hernia in Children*							
	Montupet P	J Pediatr Surg	126	1999	16.7	1.9	retrospective	Italy
35	*Thoracoscopic Repair of Tracheoesophageal Fistula in Newborns*							
	Rothenberg SS	J Pediatr Surg	108	2002	16.7	1.9	retrospective	USA
36	*Retroperitoneal Laparoscopic Vs. Open Partial Nephroureterectomy in Children*							
	El-Ghoneimi A	BJU Int	93	2003	18.3	4.8	retrospective	France
37	*Laparoscopic Vesicoureteroplasty in Children: Initial Case Reports*							
	Ehrlich RM	Urology	136	1994	19.1	1.9	case report	USA
38	*One-Trocar Transumbilical Laparoscopic-Assisted Appendectomy in Children: Our Experience*							
	D’Alessio A	Eur J Pediatr Surg	91	2002	19.8	2.3	retrospective	Italy
39	*Experience with 220 Consecutive Laparoscopic Nissen Fundoplications in Infants and Children*							
	Rothenberg SS	J Pediatr Surg	110	1998	20	1.9	retrospective	USA
40	*Laparoscopic Renal Surgery Via a Retroperitoneal Approach in Children*							
	El-Ghoneimi A	J Urol	110	1998	20	5.9	retrospective	France
41	*Laparoscopic Herniorrhaphy in Girls*							
	Schier F	J Pediatr Surg	91	1998	20.9	1.9	retrospective	Germany
42	*Laparoscopic Evaluation of the Pediatric Inguinal Hernia—A Meta-Analysis*							
	Miltenburg DW	J Pediatr Surg	109	1998	21	1.9	meta-analysis	USA
43	*Laparoscopic heminephroureterectomy in pediatric patients*							
	Janetschek G	J Urol	100	1997	23.0	5.9	retrospective	Austria
44	*Single-Blind Randomized Clinical Trial of Laparoscopic Versus Open Appendicectomy in Children*							
	Lintula H	Br J Surg	91	2001	24.2	5.7	RCT	Finland
45	*Should Laparoscopic Appendectomy Be Avoided for Complicated Appendicitis in Children?*							
	Horwitz JR	J Pediatr Surg	90	1997	25.6	1.9	retrospective	USA
46	*Is There a Role For Laparoscopic Appendectomy in Pediatric Surgery?*							
	Gilchrist BF	J Pediatr Surg	109	1992	25.7	1.9	prospective	USA
47	*Pediatric Laparoscopic Splenectomy*							
	Tulman S	J Pediatr Surg	103	1993	26.2	1.9	case report	USA
48	*Thoracoscopy in the Management of Empyema in Children*							
	Kern JA	J Pediatr Surg	103	1993	26.2	1.9	retrospective	USA
49	*Complications In Pediatric Urological Laparoscopy: Results of a Survey*							
	Peters CA	J Urol	90	1996	26.7	5.9	retrospective	USA
50	*Extramucosal Pyloromyotomy by Laparoscopy*							
	Alain JL	Surg Endosc	94	1991	30.9	3.1	retrospective	France

## Data Availability

Not applicable.
